# Right Inferior Frontal Gyrus Activation Is Associated with Memory Improvement in Patients with Left Frontal Low-Grade Glioma Resection

**DOI:** 10.1371/journal.pone.0105987

**Published:** 2014-08-26

**Authors:** Eliane C. Miotto, Joana B. Balardin, Gilson Vieira, Joao R. Sato, Maria da Graça M. Martin, Milberto Scaff, Manoel J. Teixeira, Edson Amaro Junior

**Affiliations:** 1 Department of Neurology, University of São Paulo, São Paulo, Brazil; 2 Department of Radiology, University of São Paulo, São Paulo, Brazil; 3 Centre of Mathematics, Computation and Cognition, Universidade Federal ABC, Santo Andre, Brazil; University Of Cambridge, United Kingdom

## Abstract

Patients with low-grade glioma (LGG) have been studied as a model of functional brain reorganization due to their slow-growing nature. However, there is no information regarding which brain areas are involved during verbal memory encoding after extensive left frontal LGG resection. In addition, it remains unknown whether these patients can improve their memory performance after instructions to apply efficient strategies. The neural correlates of verbal memory encoding were investigated in patients who had undergone extensive left frontal lobe (LFL) LGG resections and healthy controls using fMRI both before and after directed instructions were given for semantic organizational strategies. Participants were scanned during the encoding of word lists under three different conditions before and after a brief period of practice. The conditions included semantically unrelated (UR), related-non-structured (RNS), and related-structured words (RS), allowing for different levels of semantic organization. All participants improved on memory recall and semantic strategy application after the instructions for the RNS condition. Healthy subjects showed increased activation in the left inferior frontal gyrus (IFG) and middle frontal gyrus (MFG) during encoding for the RNS condition after the instructions. Patients with LFL excisions demonstrated increased activation in the right IFG for the RNS condition after instructions were given for the semantic strategies. Despite extensive damage in relevant areas that support verbal memory encoding and semantic strategy applications, patients that had undergone resections for LFL tumor could recruit the right-sided contralateral homologous areas after instructions were given and semantic strategies were practiced. These results provide insights into changes in brain activation areas typically implicated in verbal memory encoding and semantic processing.

## Introduction

Previous studies have demonstrated the participation of the prefrontal cortex (PFC) in executive strategic processes and episodic memory [1], [2], [3], [4], [5], [6], [7], [8], [9], [10]. In patients with acquired focal frontal lobe lesions, including cerebral tumors, episodic memory impairments are usually secondary to deficits in attention, working memory, strategy creation, the inhibition of competing recollections, and the monitoring of ongoing cognitive activity that can affect encoding, storage, and retrieval processes [1], [3], [7]. These impairments can be identified by measures of free recall, and particularly by word list tasks. Patients with PFC lesions tend to recall words in an unsystematic way and have difficulties linking words by meaning associations to facilitate encoding [1], [7], [10].

Neuroimaging studies suggest that successful verbal memory recall is associated with increased activation in the left inferior prefrontal cortex (IPFC), dorsolateral prefrontal cortex (DLPFC), and lateral prefrontal cortex (LPFC) during encoding in healthy adult subjects [3], [4], [5], [6]. An issue that has not been fully addressed is the extent to which the neural systems that underlie episodic memory encoding can be modified by strategy application in patients with frontal lobe lesions in relevant encoding areas, including the IPFC, DLPFC, and orbitofrontal cortex (OFC). This issue is highly relevant for the understanding of neural mechanisms that underlie memory encoding and improvements following interventions in patients with injuries to these specific areas. Neuroimaging studies that have investigated this particular subject are scarce. In patients with traumatic brain injury (TBI), left ventrolateral prefrontal cortex (VLPFC) activation predicted success in a 12-session group cognitive rehabilitation, and decreased activity in the left DLPFC during verbal memory encoding was related to a functional breakdown in the connectivity between these brain regions [11], [12]. Recently, we reported increased activation during verbal memory encoding in the left IPFC and precentral gyrus (PCG) after instructions and training were given in the application of semantic strategy to patients with circumscribed acquired brain lesions to the left DLPFC [10]. These activations were suggestive of recruitment of preserved perilesional areas.

Despite these previous studies, to the best of our knowledge, there have been no investigations regarding which brain regions are recruited during verbal memory encoding in patients with more extensive lesions to the left frontal lobe (LFL), including crucial memory encoding areas such as the left IPFC, DLPFC, OFC, and PCG. In addition, it is unclear whether these patients are able to improve their memory performance after instructions and practice with semantic strategies. Another issue relates to the type of lesion reported in the previous studies. Traumatic brain injury is a heterogeneous condition with a wide range of anatomical distribution, diffuse axonal injury and cognitive deficits. Patients with slow-growing tumors, particularly low-grade gliomas (LGG), have been studied over the last decade as a model of brain plasticity and functional reorganization [13], [14], [15]. The presence of discrete deficits, or the lack of cognitive and behavioral deficits, at the beginning of their appearance despite the involvement of eloquent regions have been explained by their long histories and consequent recruitment of compensatory areas [14], [15]. As a result, many patients with LGGs that infiltrate the frontal lobes, particularly in the left hemisphere, do not present with significant language or other cognitive disorders [14]. However, there is no data available regarding which brain areas are involved during episodic memory encoding in this population, or indeed whether these patients can improve their memory performance after receiving instructions and being able to practice efficient strategies.

In the present study, we investigated the neural correlates of episodic verbal memory encoding and the effects of a semantic strategy application on the brain activation of patients with extensive LFL lesions due to LGG resections. Based on previous findings with regards to tumor-induced plasticity, we hypothesized that a contralateral pattern of activation would be expected as their left encoding related areas had been resected. We also included healthy control subjects in order to compare the performed of both groups in terms of brain activation and behavioral performance.

## Methods

### Participants

**Figure 1:**
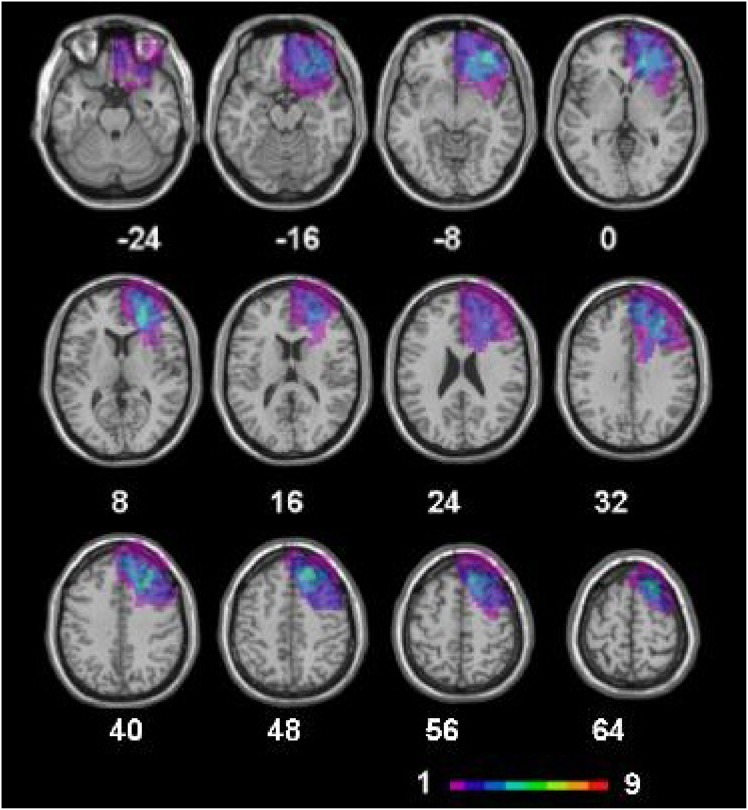
Overlap lesion maps for patients with LFL lesions. Lesions are projected on the same axial slices on a standard brain template. The color bar indicates the number of overlapping lesions. Left hemisphere is on the right side of the figure.

Patients were selected among those referred to the Neuro-Oncology outpatient clinic at the Department of Neurology, University of Sao Paulo. Nine right-handed patients with extensive and specific LFL lesions due to a subtotal (more than 90%) or total LGG resection of the OFC, IPFC, DLPFC, superior, and PCG and 15 right-handed healthy controls were included. Lesion topography and size were evaluated by blinded radiologists using fluid attenuated inversion recovery (FLAIR) and high-resolution T1 sequences (see lesion characteristics in Table 1 and the overlap lesion maps in Figure 1).

**Table 1:** Age, lesion size and location for each individual LFL patient.

Subjects	Age	Yearsaftersurgery	Etiology	Lesionsize (cc)	Damage location inthe left hemisphere
P1	27	1	Low GradeGlioma	16.02	MFG, extending laterally to SFGand to the anterior face of the preCG
P2	30	1	Low GradeGlioma	74.94	OrbG and rectus gyrus, extending toanterior and posterior portions of SFGand MFG, IFG, IFGpt and CingG
P3	52	2	Low GradeGlioma	3.26	limited damage to the medialportion of SFG
P4	45	5	Low GradeGlioma	38.3	posterior MFG, extending to the IFGand SFG and the anterior border ofthe preCG
P5	26	3	Low GradeGlioma	0.4	lOrgG, IFGpo
P6	42	3	Low GradeGlioma	23.08	pOrbG, lOrbG, IFGpo extending to insula
P7	42	0	Low GradeGlioma	86.86	anterior and basal portions of the SFG,IFG and MFG
P8	50	11	Low GradeGlioma	81	OrbG, IFG and medial portion of the SFG
P9	36	0	Low GradeGlioma	68.18	aOrbG, mOrbG, medial portion of SFG,anterior border of the MFG, CingG

Abbreviations: MFG, middle frontal gyrus, SFG, superior frontal gyrus; OrbG, orbital frontal gyri; lOrbG, lateral orbital frontal gyrus; pOrbG, posterior orbital frontal gyrus; aOrbG, anterior orbital frontal gyrus; mOrbG, middle orbital frontal gyrus; IFGpo, inferior frontal gyrus pars orbitalis; IFGpt, inferior frontal gyrus pars triangularis; CingG, cingulate gyrus; preCG, precentral gyrus.

Patients who had undergone a tumor resection outside the LFL area and those who had an etiology other than LGG were excluded. Included patients had not received any drug treatment, chemotherapy, or brain radiation within the previous 6 months or during the period of the study. In addition, they were free from other neurological or psychiatric disease and were non-aphasic, as tested by neurological and neuropsychological evaluations. Time since surgery ranged from 12 months to 10 years (mean = 2.65 years, SD = 2.40). All participants in the study underwent clinical and neuropsychological assessments. These included Full IQ (WAIS-III) [16], short-term memory (Digit Span-WAIS-III) [16], episodic memory (Warrington Recognition Memory Test [17] and the Rey Auditory Verbal Learning Test) [18]. The study was approved by the ethics committee of the Department of Neurology, Hospital das Clinicas, University of São Paulo (CAPPesq 271/01) and the patients signed a written informed consent form prior to their inclusion. All patients had preserved mental and motor capacity to provide and sign the informed consent form.

### Study design and functional MRI paradigm

**Figure 2:**
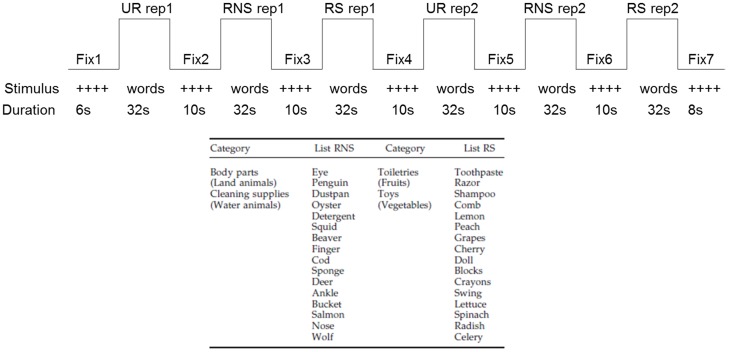
Experimental paradigm. Experimental conditions: fixation baseline (+); unrelated words (UR); related-structured words (RS); related-non-structured words (RNS). There were three runs, each run with 48 words and total number of words in the first or second fMRI session = 144 words.

The current paradigm was based on the California Verbal Learning Test (CVLT) [19], adapted from a previous PET study [5] and validated in healthy adults using fMRI [6]. Participants were scanned during the encoding of word lists under three different conditions (blocks) and three runs before and after receiving instructions and a brief period of practice to apply the semantic strategies. This unique paradigm allows for the greater or lesser use of semantic organization according to the specific condition. For each run, the three blocks consisted of 16 unrelated words (UR), 16 related-non-structured words (RNS), 16 related-structured words (RS) and a fixation baseline (+) were presented twice, and each word was presented every two seconds (see Figure 2). For the UR condition, the words presented did not share any semantic relationship and the use of a semantic strategy would prove extremely difficult. For the RNS condition, the words were related in terms of categories (e.g., fruits, land animals, etc.); however, they were randomly presented or appeared in a mixed order. For the RS condition, the words were related in terms of categories and presented grouped together into categories (see Figure 2 for sample word lists). The word lists were generated from 32 categories of words with four words in each category, balanced for word length; their validity in prompting significant differences in semantic clustering had been tested in previous studies (see [5], [6] for more detailed descriptions).

Each subject was scanned twice: before (1^st^ fMRI session) and after (2^nd^ fMRI session) with a 30-min off scan practice with instructions to apply the semantic organizational strategies. All stimuli were visually presented on a screen and synchronized with the scanner via an optic relay triggered by the radiofrequency pulse. The presentation order of the words in each list was randomized and conditions were counterbalanced across participants. In the 1^st^ fMRI session, subjects were instructed to look at the words presented onto the screen and to try to remember as many words as they could, with no specific instructions to apply any strategy. They were informed that their memory for the words would be tested later on. Following each run, participants were instructed to produce as many words as they could remember in any order. At the end of the 1^st^ fMRI session, they were taken to a different room and given a 30-min period of instructions and practice to apply semantic organizational strategies to a set of five different word lists. Subjects were instructed to organize the words into categories and to retrieve them according to their category. All participants were able to learn and apply the semantic strategies to at least three word lists by the end of the 30-min practice period. Afterwards, they were scanned for the second time during memory encoding and instructed to apply the semantic organizational strategies to the novel word lists. Semantic clustering index scores were defined as the consecutive recall of two words from the same category. They reflected the proportion of clustered responses out of the total possible clusters defined as follows: clusters/(words recalled-categories recalled). The serial clustering score was defined as follows: clusters/(words recalled–1).

### Image acquisition and processing

Gradient echo planar imaging (EPI) data were acquired on a GE Signa 1.5T system (General Electric, Milwaukee WI, USA). One-hundred and twenty-eight T2*-weighted images (BOLD contrast) were obtained over 5 minutes (for each of the six runs, half at each fMRI session) in 15 axial non-contiguous 7-mm thick planes (inter-slice gap 0.7 mm, in-plane resolution 3.125×3.125 mm) parallel to the intercommissural (AC-PC) line with TE 40 ms and TR 2 s.

Lesions were initially masked in order to avoid incorrect spatial deformation during image registration processes. A neuroradiologist (blinded to the study main goals, including the patient group subdivision) manually traced all lesions using the high resolution SPGR and FLAIR images. Individual binary lesion masks were then applied to the EPI data using cost-function masking in FSL 4.1 (FMRIB Analysis Group, www.fmrib.ox.ac.uk/fsl). A lesion overlay map was constructed using areas within the lesions color-coded according to the frequency of voxels set to 1. A masking procedure to constraint statistics only to voxels that represented preserved regions common to all patients was used during statistical analysis. An image was created from the overlay plot overlapping all of the lesions. Then, this overlay image was subtracted from the patients’ group image. The resultant image was used as a region of interest in the pre-threshold masking for both the within sessions comparison in the patients group and in the comparison between patients and controls.

FSL package (version 4.1, FMRIB Analysis Group, www.fmrib.ox.ac.uk/fsl) was used to analyze the fMRI data. Preprocessing included movement correction (MCFLIRT), spatial smoothing and spatial normalization to a MNI-152 template with a 12 DoF affine registration (FLIRT).

In a first-level analysis, a general linear model (GLM) approach was used to obtain statistical activation maps. The design matrix was composed of regressors according to the experimental conditions (RNS, RS, and UR). For each condition, the hemodynamic response function (HRF) in GLM was adopted using a gamma function (SD = 3 s, mean lag = 6 s) and its first derivative. Single subject statistical maps representing the average activation within the three runs collected in each fMRI session (1^st^ and 2^nd^) were created using a fixed-effects model. Z-statistic images derived from the FSL were thresholded using clusters of voxels determined by z-voxels >2.3 and a corrected cluster significance of 0.05.

### Comparison within sessions

In a second level analysis, a mixed-effect model was used to assess BOLD activation changes across sessions using the following contrasts [(RNS>fixation)_fMRI 1_< (RNS>fixation)_fMRI 2_], [(RS>fixation)_fMRI 1_< (RS>fixation)_fMRI 2_], [(UR>fixation)_fMRI 1_< (UR>fixation)_fMRI 2_] and vice versa, for healthy subjects and LFL patients independently.

### Comparison between groups

To investigate whether the use of the semantic strategy affected brain activation differently between patients and controls, the interaction between group and time was assessed by a t contrast of the positive and negative interaction effects, according to the following contrasts:

[(RNS>fixation)_fMRI 1_<(RNS>fixation)_fMRI 2_]_LFL_>[(RNS>fixation)_fMRI 1_<(RNS>fixation)_fMRI 2_]_Control_ and vice versa.

**Correlation between performance and activation.** At the whole brain level, we tested correlations between changes in behavioral memory measures and brain activation across sessions. The association between changes in the semantic clustering index and in the number of words recalled for each participant (fMRI 2– fMRI 1, Δ behavior), and the corresponding change in BOLD activation (Δ BOLD session) were analyzed using a linear regression analysis. To this end, a map was created in a second-level analysis by subtracting the map associated with the encoding of the related non-structured list (RNS>fixation) at the fMRI 1 session from that of the fMRI 2 session for each participant. Finally, to verify whether there was a difference in brain-behavior correlation patterns between groups, the interaction group*Δ behavior session was examined.

In the second approach, which was aimed to characterize brain activation associated with the strategy application at the individual patient level, parameter values estimated from the GLM analysis that reflected baseline and change in fMRI activation were computed for each of the perilesional masks. As there is no consensus in the literature about the spatial parameters that are used to define the perilesional cortex, we used the information provided by recent published studies on perilesional activation after the recovery of stroke-induced aphasia [20], [21]. Individual perilesional masks were traced if they expanded 10 mm beyond the rim of the lesion. The perilesional region of interest was masked with a grey matter mask originated from FSL first level analysis in order that beta-values were only obtained from grey matter. Mean beta-values of the individual brain images representing changes in fMRI activation were used as predictor variables to estimate changes in behavior. This analysis was completed using SPSS 17.0.0.

## Results

### Clinical and behavioral data

Table 2 presents the demographic characteristics of the patients and healthy controls. The age and educational level of the two groups were matched. Their performance did not differ on the cognitive tests, with the exception of the LFL group who performed in the low-average range on the immediate and delayed recall of the RAVLT. This non-related word list test measures verbal episodic memory with the involvement of executive functions. This finding is consistent with previous studies in patients with tumors in the frontal lobe region that show reduced memory recall as a consequence of executive difficulties [15], [22]. It should be noted that the patients did not present with aphasia or other language deficits.

**Table 2:** Clinical characteristics of the LFL and healthy control subjects. Results are expressed as mean (SD).

	Healthy controls (n = 15)	LFL Group (n = 9)
Education	7.93 (3.49)	8.67 (3.64)
Full IQ (WAIS-III)	96.87 (6.63)	99.67 (9.95)
Digit span percentile (WAIS-III)	42.20 (17.19)	41.67 (20.29)
Verbal recognition percentile (WRMT)	43.0 (30.16)	39.5 (29)
Visual recognition percentile (WRMT)	54.33 (23.13)	51.45 (28.89)
RAVLT immediate total recall percentile	43.13 (4.3)	19.2 (8.2)
RAVLT delayed recall percentile	39.3 (1.3)	19.8 (2.3)

WAIS-III = Wechsler Adult Intelligence Scale; RAVLT = Rey Auditory Verbal Learning Test; WRMT = Warrington Recognition Memory Test.

Free recall scores in each of the three encoding conditions were analyzed with a two (group: controls vs. LFL patients) by two (time: before vs. after) repeated measures ANOVA (Table 3). For the UR condition, no main effects between groups (F = 1.22; p = 0.574), or time (F = 1.22; p = 0.440) were observed (Table 2). In the RNS condition, the repeated measures ANOVA showed a significant time effect (F = 1.22; p = 0.032), which indicated that both groups had improved their recall and no significant differences were found between groups after the instructions were given about the semantic strategy. For the RS condition, there was a significant group x time difference (F = 1.22; p<0.001) with better recall scores for the controls in comparison to the LFL patients, only before the instructions to use the semantic strategy. The repeated measures ANOVA for the clustering index scores showed a significant difference between the groups only before the directed instructions were given to apply the strategies (F = 1.22; p<0.008).

**Table 3:** Memory performance measured during the 1^st^ and 2^nd^ fMRI sessions for the healthy controls and LFL group.

	Controls		LFL Group	
	1^st^ fMRI	2^nd^ fMRI	1^st^ fMRI	2^nd^ fMRI
Free recall				
UR	6.00 (0.77)	6.26 (0.96)	5.44 (1.00)	5.62 (1.24)
RNS	12.46 (1.37)	15.33 (1.60)	9.44 (1.77)	13.82 (2.06)
RS	17.53 (1.62)	20.01 (1.54)	13.44 (2.09)	18.66 (1.98)
Clustering index	0.33 (0.86)	0.43 (0.05)	0.21 (0.09)	0.37 (0.09)

### Functional MRI results

#### Effects of task repetition on fMRI activation

A similar approach used by Belleville et al. (2011) [23] was adopted to evaluate the fMRI task repetition effect on brain activation before the directed instructions to apply the semantic strategy were given. The activations related to the first and second runs acquired in the first fMRI session were compared within each group separately, in order that each subject was used as its own control. No significant brain activation changes between the runs were observed in controls or patients with LFL lesions, which suggested that repeating the task without the instructions and practicing the strategies did not produce measurable repetition or practice effects on brain activation.

#### Brain activation during verbal memory encoding after instructions for the semantic strategy were given

**Figure 3:**
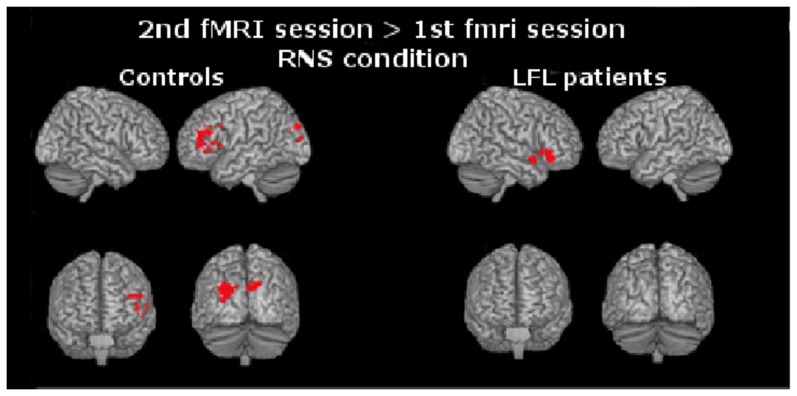
Increased activation in the 2ndfMRI session for the RNS condition. In healthy adult controls, changes were seen in the left prefrontal cortex, including the inferior and middle frontal gyri, intraparietal sulcus, cuneus and precuneus. In patients with LFL, statistical significant changes were seen in the contralesional hemisphere, mainly in the right inferior frontal gyrus, extending to the superior temporal gyrus and insula.

In healthy subjects, no significant brain response was observed in the contrast 1^st^ fMRI>2^nd^ fMRI session (UR, RS, RNS) or in the contrast 2^nd^>1^st^ session for either the UR or RS conditions. For the RNS condition, healthy participants exhibited increased activation in the 2^nd^ fMRI session in the left hemisphere, including the IFG (BA 45/46), MFG (BA 11), PCG (BA 6), intraparietal sulcus (BA 39), right precuneous (BA 7), and bilateral cuneus (BA 39/18). Figure 3 shows the brain activation areas for the RNS condition and Table 4 describes the coordinates in the MNI space and peak activation values of clusters with significant changes in the BOLD signal of healthy volunteers.

**Table 4:** Coordinates in MNI space and peak activation values of clusters with significant changes in BOLD signal of healthy volunteers when comparing the 1^st^ fMRI and 2^nd^ fMRI sessions for the contrasts RNS>baseline, UR>baseline and RS>baseline.

Contrast	Side	Region	BA	MNI Coordinates					Peak		Cluster size(number of voxels)	Clusterp- values
				x	y	z			Z stat			
2^nd^>1^st^ fMRI												
RNS>baseline	L	Inferior frontal gyrus	45	–48	14	28			3.72		3475	<0.001 (cluster 1)
	L	Inferior frontal gyrus	46	–44	40	8			3.6			
	L	Middle frontal gyrus	11	–40	40	10			3.63			
	L	Precentral gyrus	6	–40	2	34			3.37			
	L	Intraparietal sulcus	39	–32	–58	36			3.81		2923	<0.001 (cluster 2)
	L	Cuneus	39	–8	–82	30			2.73			
	R	Cuneus	18	6	–80	28			3.67			
	R	Precuneus	7	12	–74	28			3.52			
									
UR>baseline			No activation									
RS>baseline			No activation									
									
1^st^>2^nd^ fMRI												
RNS>baseline			No activation									
UR>baseline	No activation											
RS>baseline			No activations									

In the LFL group, no significant differences were observed in the contrast 1^st^ fMRI>2^nd^ fMRI session (UR, RS, RNS) or in the contrast 2^nd^>1^st^ session for the UR or RS conditions. During the memory encoding of words for the RNS condition, LFL patients demonstrated increased activation in the right hemisphere involving the IFG (BA 45), MFG (BA 11), superior temporal gyrus (BA 38), and insula (BA 13). These regions are similar to those observed in the same comparison in healthy controls, but in the contralateral homolog area in the right hemisphere (see Figure 3 and Table 5 for coordinates in the MNI space).

**Table 5:** Coordinates in MNI space and peak activation values of clusters with significant changes in BOLD signal of patients with LFL lesions when comparing the 1^st^ fMRI and 2^nd^ fMRI sessions for the contrasts RNS> baseline, UR> baseline and RS>baseline.

Contrast	Side	Region	BA	MNI Coordinates			Peak	Cluster size (number of voxels)	Cluster p-values
				x	Y	z	Z stat		
2^nd^>1^st^ fMRI									
RNS>baseline	R	Inferior frontal gyrus	45	48	30	–6	3.32	1306	<0.001 (cluster 1)
	R	Middle frontal gyrus	11	34	34	–12			
	R	Superior temporal gyrus	38	54	8	–12			
	R	Insula	13	44	6	–12			
									
									
UR>baseline			No activation						
RS>baseline			No activation						
1^st^>2^nd^ fMRI									
RNS>baseline			No activation						
UR>baseline			No activation						
RS>baseline			No activation						

**Figure 4:**
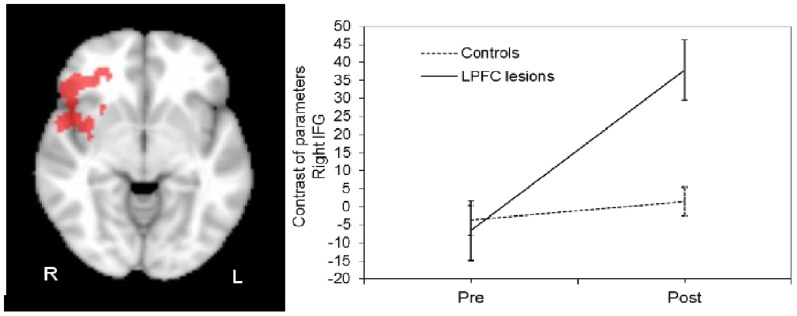
Axial slice showing the cluster with local maxima in right IFG (MNI coordinates 48, 28, −6). Group x fMRI Session interaction was significant (voxel Z>2.3, cluster corrected p<0.001) for the contrast RNS>baseline. The graph shows the amplitude of BOLD responses in right inferior frontal gyrus (mean and standard error). Functional MRI session-related differences in the contrast of parameters estimate values are greater for LFL patients compared to controls.

As both controls and LFL groups exhibited fMRI session-related differences in brain activation for the contrast RNS>baseline, we tested the interaction effect group x time. A significantly positive interaction of session and group was found in a cluster with local maxima in the right IFG (BA 45/46; voxel Z>2.3, cluster-corrected p<0.01; Figure 4). A plot showing the mean magnitude estimates of activity in the significant cluster for each session indicated the nature of the interaction effect. The activation in the right IFG increased across sessions for the LFL patients and remained stable for the controls. There were no significant correlations between changes in behavioral memory measures and brain activation across sessions at the whole brain level.

#### Perilesional functional MRI activation and memory performance

No significant results were found in the regression analyses conducted to examine the relationship between strategy-related increases in free recall and changes in brain activation in the perilesional region-of-interest (ROI) for the RNS condition in the LFL group.

## Discussion

The purpose of this study was to investigate the neural correlates of memory encoding and the effects of the instructions to apply semantic strategies on the brain activation of patients with extensive LFL lesions due to LGG resection compared to healthy controls. Both groups had improved memory performance during the second fMRI session after instructions on how to use the semantic organizational strategies were given. In addition, the differences between groups in terms of memory and strategy index scores disappeared during the second fMRI session after they received these instructions. These findings indicate that, despite the presence of widespread lesions in relevant areas for verbal memory encoding, patients with LFL excisions were able to learn and increase the application of efficient strategies in order to improve their memory recall. Similar behavioral results were found in previous studies with healthy adult and elderly subjects [5], [6], [9] and in a recent study in patients with more circumscribed heterogeneous lesions to the left DLPFC and OFC [10].

In terms of neuroimaging results, different patterns of brain activation were found for the two groups, both within and between groups. These results are discussed separately in the following sections.

### Brain activation during verbal memory encoding after semantic strategy application in healthy subjects

For the RNS condition, which is the one in which semantic processing and active organization in working memory are most in demand, healthy adult controls showed increased activation in the 2^nd^ fMRI session in the IFG (BA 45/46), MFG (BA 11), PCG (BA 6), intraparietal sulcus (BA 39) in the left hemisphere, right precuneus (BA 7), and bilateral cuneus (BA 39/18). These findings corroborate other studies that included healthy young and older adults, which showed increased activation of the left PFC, parietal regions, and precuneus during verbal memory encoding tasks with higher executive processes load engaging strategic, working memory, and monitoring of responses [3], [5], [6], [9]. There were no significant changes in brain activation for the UR or RS conditions in the contrast 2^nd^>1^st^ fMRI or in the 1^st^>2^nd^ fMRI session for the UR, RS or RNS conditions. These results suggest that the effect of the increased use of strategy for brain activation was specific to the RNS condition in the 2^nd^ fMRI session after the instructions on how to apply the strategies were given. Higher-level control operations, such as working memory and executive memory-related functions, might have enabled subjects to maintain and organize words from distinct categories into distinct memory traces to be further recalled [5], [6], [9], [10], [24], [25].

### Brain activation during verbal memory encoding after semantic strategy application in LFL patients

LFL patients showed increased levels of activation for the RNS condition in the contrast 2^nd^>1^st^fMRI session in the right IFG (BA 45/46), MFG (BA 11), superior temporal gyrus (BA 38), and insula (BA 13). These brain-activated areas are similar to those found in the within-group comparison between sessions in healthy controls for the RNS condition, but in the contralesional area on the right hemisphere. These findings suggest that, despite damage to relevant areas that support episodic verbal memory encoding and semantic strategy applications in the LFL group, right contralesional homologous areas were functionally recruited after the instructions on how to apply efficient semantic strategies were given. To our knowledge, this is the first time that these findings have been demonstrated in patients with large LFL excisions. The ANOVA analysis of group x time interactions corroborated the contralesional activation in the LFL patients by showing a significant effect in the right inferior frontal gyrus (BA 45/46), with increased activation in the 2^nd^ fMRI session (versus the first) during the RNS condition in LFL patients, while a stable activation over sessions was observed in controls. This finding argues in favor of the functional compensation or recruitment of the contralesional right IFG preserved area in these patients.

It is well known that the left IFG supports the use of semantic processing in verbal memory encoding tasks [5], [24], [25]. As mentioned earlier, the patient sample included in our study presented with LGG tumors in the LFL and, therefore, it is possible that due to the long history of the lesion, some cognitive compensation processes had already taken place, including language reorganization [13], [14], [15]. Although it is difficult to argue for long-term brain plasticity due to the brief period given to practice the semantic strategies, we showed that patients with extensive LFL lesions had improved memory performance, and this improvement was associated with the recruitment of the right contralesional IFG region.

An issue that could be raised is to what extent the brain activation changes demonstrated by the LFL patients and healthy controls were related to the repeated exposure to the word lists across each run instead of the instructions to apply the strategies. To this end, we used a similar approach to the one that Belleville et al. [23] adopted to examine the fMRI task repetition effect on brain activation. We compared the activation of the first and second runs acquired in the 1^st^ fMRI session before the instructions on how to use the strategies were given within each group separately. Therefore, each subject was used as its own control. There were no significant changes in brain activation for the controls or patients with LFL lesions, which suggested that repeating the task without the instructions and practicing the semantic strategies did not produce measurable or repetition effects on brain activation.

At least three important aspects must be taken into account when considering the findings of the current study. Firstly, the detection of possible left hemisphere activation in some of the patients was prevented by the masking procedure applied to the fMRI statistics (i.e., constraint statistics were only applied to voxels that represented preserved regions common to all patients). On the visual inspection of individual statistical maps, we observed that three patients had clusters of significant activation changes between sessions in the left hemisphere. These clusters were located in areas that were also found in the average group image (fMRI 2>fMRI 1) of the control group. Two patients had more anterior and inferior resections in the LFL, which may explain the activation in the left hemisphere. The third patient had a resection that included the superior and medial frontal gyri and the subcortical white matter, with activations found bilaterally, and was dominant on the right hemisphere with few clusters on the left hemisphere. Nevertheless, these findings enable only a limited interpretation, and should be addressed in future studies with a larger group of patients and in specific subgroups that consider the cortical and subcortical areas. Secondly, the non-significant associations between the activation change in perilesional regions and the strategy-related improvement in free recall examined in a whole-brain analysis might be related to the variability in lesion extent and, consequently, to the reduced number of preserved voxels in the left hemisphere due to the masking procedure adopted. Regarding the perilesional ROI analysis, in some cases, especially in those patients with large volume lesions, the perilesional region encompassed portions of cortex that may not be functionally relevant to the task, which was also interfered in the lack of significant brain-behavior correlations. Thirdly, our data lacked the power for an in-depth analysis of inter-relationships between variables, including the correlation involving behavior and imaging results, probably due to the small sample size. This hypothesis should be further investigated in studies with larger patient groups.

Despite these limitations, the current results support the use of fMRI to identify brain activity related to memory encoding and strategy application in patients with LGG. They also provide relevant information regarding changes in brain activation areas that are typically implicated in verbal memory encoding and semantic processing. In the present study, we showed that a brief period of practicing semantic strategies in patients with extensive LFL lesions is sufficient to produce differences in behavior and brain responses. Future studies that recruit larger patient groups and involve longer periods of interventions are necessary to corroborate these findings and investigate the underlying mechanisms of neuroplasticity and functional brain reorganization.
